# Antiplatelet mechanism of an herbal mixture prepared from the extracts of *Phyllostachys pubescens* leaves and *Prunus mume* fruits

**DOI:** 10.1186/s12906-017-2032-5

**Published:** 2017-12-19

**Authors:** Eunjung Son, Seung-Hyung Kim, Won-Kyung Yang, Dong-Seon Kim, Jimin Cha

**Affiliations:** 1 KM Convergence Research Division, Korea Institute of Oriental Medicine, 672 Yuseong-daero, Yuseong-gu, Daejeon, 305-811 Republic of Korea; 2 Institute of Traditional Medicine and Bioscience, Daejeon University, Daejeon, 300-716 Republic of Korea; 3 Department of Microbiology, Faculty of Natural Science, Dankook University, Cheonan, Chungnam, 330-714 Republic of Korea

**Keywords:** Bamboo leaf, *Phyllostachys pubescens*, *Japanese apricot* fruit, *Prunus mume*, Anti-platelet aggregation, Anti-thrombosis

## Abstract

**Background:**

Bamboo (*Phyllostachys pubescens*) leaves and Japanese apricot (*Mume fructus*) fruit are traditionally recognized to be safe herbs broadly used for food and medicinal purposes in Southeast Asia. Our group previously explored their antiplatelet effects. This study was designed to confirm inhibition effects of PM21 (a 2:1 mixture of bamboo leaf extract and Japanese apricot fruit extract) on platelet aggregation and evaluate its potency to use as an herbal remedy to prevent and/or treat the diseases caused by platelet aggregation and thrombus formation.

**Methods:**

Washed platelets were prepared and platelet aggregation was induced by adding 5 μg/mL collagen. Anti-platelet effects of PM21 (75 mg/kg, 150 mg/kg, and 300 mg/kg for ex vivo and in vivo assays, and 50, 100, 200 μg/mL for in vitro assays) were evaluated. In ex vivo assays, PM21 was orally administered to rats daily after overnight fasting for 3 days and blood was collected 1 h after the final treatment*.* In vivo antithrombotic effect of PM21 was observed from a carrageenan induced mouse tail thrombosis model.

**Results:**

In ex vivo assay, PM21 inhibited platelet aggregation significantly. PM21 showed a strong antithrombotic effect by reducing significantly the length of mouse tail thrombus. PM21 increased intracellular cAMP level and reduced the release of ATP, TXA_2_, and serotonin. PM21 also reduced intracellular concentration of calcium ion, fibrinogen binding to integrin α_IIb_β_3_, and phosphorylation of ERK2, p38, PLCγ2, and PI3 K.

**Conclusions:**

PM21 showed remarkable inhibitory effects on platelet aggregation and thrombus formation. Its inhibitory function seems to influence on GPVI binding to its ligand and subsequent initiation of a signaling cascade that involves activation of effector proteins and secretion of effector molecules, such as ATP, TXA_2_, serotonin, and Ca^2+^. PM21 also appears to exert its anti-platelet effect by deactivation of *ERKs* activation pathway as well as inhibition of fibrinogen binding to integrin α_IIb_β_3_.

## Background

When a blood vessel is injured platelets and fibrin play a major role to form blood coagulation to prevent blood loss from the damaged vessel. However this blood coagulation can cause a various kinds of venous or arterial pathogenesis, such as congestion, ischemia, and necrosis that can subsequently result in some serious cardiovascular diseases, such as a stroke [1] myocardial infarction [2] and arteriosclerosis [3, 4]. The debris of thrombus can be detached from the site of formation to travel the circulation system as an embolus causing the development of thrombosis into thromboembolism. These thrombus formation and embolic events are significant causes of many cardiovascular diseases [5].

Platelet aggregation is an important procedure for effective thrombus formation following the adhesion of platelets to the site of injury [6]. The platelets can bind to collagen and initiate cellular activation processes. Platelet collagen receptors are grouped on the basis of their interaction with collagen. Platelet GPVI is the major platelet collagen receptor in the formation of platelet aggregates on collagen surfaces under blood flow [7].

A number of agonists activate platelets by binding to specific surface receptors. Activated platelets release the stored granule contents that include ADP (adenosine diphosphate), serotonin, PAF (platelet-activating factor) and synthesize TXA_2_ (thromboxane A_2_), a potent platelet activator, via prostaglandin H_2_ and arachidonic acid [8]. Released granule contents, in turn, activate other platelets [6] and lead to a series of downstream events that finally cause to elevate intracellular concentration of calcium ion [9]. Increased intracellular concentration of calcium ion results in a number of structural and functional changes. Changes in the shape of platelets allow them to interact with each other to form aggregates [10].

*Phyllostachys pubescens* is a giant timber bamboo native to China and widely distributed in tropical and subtropical zones of the world. Various parts of this bamboo have been used as a source of traditional medicine in many countries. Bamboo leaves have been an important ingredient in Chinese traditional prescriptions, and its therapeutic properties have long been practiced to treat many symptoms including inflammatory and cardiovascular lesions for thousands years [11, 12]. Therapeutic effects of *Phyllostachys pubescens* leaves on cardiovascular lesions, such as ischemia [13], myocardial infarction [14], and thrombus formation [15] have been reported.

*Prunus mume* is an Asian tree species commonly known as Japanese apricot. Its fruit has long been used as a traditional medicine and healthy food in East Asian countries [16]. It has been reported that *Prunus mume* fruit has antibacterial [17, 18], antioxidant [19], antivirus [20], antitumor [21], immune enhancing [16] and hypouricemic [22] effects.

We previously reported that mixtures of *Phyllostachys pubescens* leaves (PL) and *Prunus mume* fruit (MF), especially at the ratio of 2:1 (PM21), inhibited platelet aggregation and thrombus formation more efficiently than PL or MF alone, appreciating the potency of PM21 to utilize for the prevention of thrombosis [15]. Finding an herbal remedy that can be easily accessed and safely consumed by common people susceptible to cardiovascular pathogenesis may offer a possible health care measure as a complementary and alternative medicine.

It may be valuable to study antithrombotic and anti-platelet mechanisms to find useful therapeutic targets to enhance the development of effective cardiovascular agents. This study was designed to elucidate major factors involved in the anti-platelet mechanism of PM21 to confirm our previous anti-platelet mechanism study [23]. We investigated the action mechanism of PM21 with respect to platelet activation in due course of platelet aggregation, focusing on the collagen receptor, GPVI (glycoprotein VI) signaling pathway and *ERKs* (extracellular signal-regulated kinases) activation pathway.

## Methods

### Materials

Collagen was obtained from Chrono-Log Co. (Havertown, PA, USA). Aspirin, fibrinogen, dimethyl sulfoxide (DMSO), and Fura-2/AM were obtained from Sigma Chemical Co. (St. Louis, MO, USA). Antibodies of phospho-p38, p38, phospho-SAPK/JNK, phospho-PI3 K (p85), class I PI3 K and isoforms, and β-actin were purchased from Cell Signaling (Beverly, MA, USA). ATP (adenosine triphosphate) assay kit was purchased from Biomedical Research Service Center (Buffalo, NY, USA). TXB_2_ enzyme immunoassay (EIA) kit was purchased from Cayman Chemical (AnnArbor, MI, USA). Fibrinogen Alexa Fluor 488 conjugate was purchased from Molecular Probes (Eugene, OR, USA).

### Preparation of plants extracts

Bamboo (*Phyllostachys pubescens*) leaves were collected from Nanjing, China, on November 28, 2012, and left to dry in storage space at room temperature. Japanese apricot fruits were collected in Gwangyang, Korea, on June 22, 2012. The voucher specimens were identified by comparing their shape, position, thickness, and color with the specimen in the Basic Herbal Medicine Research Group at Korea Institute of Oriental Medicine. The specimens were identified by Eunjung Son and Dong-Seon Kim and authenticated by Classification and Identification Committee of the Korea Instituted of Oriental Medicine (KIOM). Authenticated voucher specimens (BL-20120727; MF-20120725) were deposited in the Herbarium of Korea Institute of Oriental Medicine. Unripe fruits were dried at 55 °C in a convection oven until their skins turn to black. 1 kg each of bamboo leaves and Japanese apricot fruits were pulverized and extracted individually with 14 L of 80% (*v/v*) ethanol in water for 5 h at 82 °C. Two extracts were filtered and then evaporated under a reduced pressure in a rotary evaporator (N-1000 S; EYELA, Tokyo, Japan). 92 g of bamboo leaf extract and 410 g of Japanese apricot fruit extract were harvested. Bamboo leaf extract and Japanese apricot fruit extract were mixed at the ratio of 2:1, respectively to obtain the herbal mixture preparation, PM21.

### Experimental animals

Male Sprague–Dawley rats weighing from 240 to 250 g and male ICR mice (6 weeks age and 20 ~ 23 g) were obtained from Daehan Biolink Co. Ltd. (Eumsung, Republic of Korea), maintained in a standard laboratory animal facility, and randomly distributed experimental animals to each experimental group. The rats and mice had been acclimated for 2 weeks before the experiment started and their consumption of food and water was noted. This study was approved by the Animal Welfare Committee of Daejeon University. All animal experiments were performed in accordance with the guidelines of the Institutional Animal Care and Use Committee of Daejeon University, Republic of Korea (DJUARB2014–48).

### Preparation of platelet-rich plasma and washed platelets

Rats were fasted overnight and euthanizated with urethane (1.25 g/kg, i.p.). Blood samples were collected from the abdominal vein of rats and transferred directly into ACD (anticoagulant citrate dextrose) solution containing 0.8% citric acid, 2.2% trisodium citrate, and 2% dextrose (*w/v*). Washed platelets were prepared as previously described [24]. PRP (Platelet-rich plasma) was obtained by centrifuging anti-coagulated blood samples at 230 ×g for 10 min. After removing red blood cells, platelets were precipitated by centrifugation of PRP at 800 ×g for 15 min and washed with HEPES buffer (137 mM NaCl, 2.7 mM KCl, 1 mM MgCl_2_, 5.6 mM glucose, 3.8 mM HEPES, and pH 6.5) containing 0.35% BSA and 0.4 mM EGTA (Ethylene Glycol Tetra-acetic Acid). The washed platelets were resuspended in HEPES buffer (pH 7.4) and adjusted to 4 × 10^8^ cells/mL.

### Ex vivo assay of platelet aggregation

Six male rats were allocated in vehicle group, positive control group, and three test groups. PM21 was orally administered daily after overnight fasting for 3 days to three test groups at the doses of 75 mg/kg, 150 mg/kg and 300 mg/kg, and aspirin was administered to positive control group at the dose of 50 mg/kg. Platelet aggregation was evaluated following the assay protocol previously described [25]. Blood was obtained by cardiac puncture and collected in a plastic flask containing 3.28% sodium citrate solution (10% blood, *v/v*) 1 h after the final treatment. PRP was prepared as described previously in this paper. Platelet aggregation was monitored by measuring light transmission with an aggregometer (Chrono-Log, Havertown, PA, USA). Washed platelets were pre-incubated at 37 °C for 2 min and then stimulated with 5 μg/mL collagen in phosphate buffer solution. The mixture was further incubated for 5 min with stirring at 170 x g and changes in light transmission were recorded and the maximal aggregation rate was observed.

### Ex vivo assay of ATP release

Rats were orally administered with different doses of PM21 daily after overnight fasting for 3 days. Blood was collected from the heart of rats by cardiac puncture after the last treatment, and washed platelets were prepared as previously described [24]. Washed platelets (3 × 10^8^/mL) were pre-incubated for 2 min at 37 °C and then stimulated with 5 μg/mL collagen. After the aggregation reaction was terminated, the cells were centrifuged and the supernatant was used for the assay. ATP release was measured with the aid of luminometer (GloMax 20/20; Promega, Madison, USA) using ATP assay kit (Biomedical Research Service Center, Buffalo, NY, USA).

### Assessment of fibrinogen binding to integrin α_IIb_β_3_

Fibrinogen Alexa Fluor 488 conjugate binding to washed platelets was quantified by flow cytometry. In this experiment, washed platelets (3 × 10^8^/mL) were pre-incubated for 2 min with various concentrations (200, 100, 50 μg/mL) of PM21 at room temperature. The platelets were then stimulated with 5 μg/mL collagen in the presence of *Ca* ^2+^ (1 mM) for 5 min, and immediately incubated thereafter with fibrinogen Alexa Fluor 488 (20 μg/mL) for 5 min, and finally fixed with 0.5% paraformaldehyde at 4 °C for 30 min. The platelets were pelleted by centrifugation at 2000×g at 4 °C and resuspended in 500 μL PBS (Phosphate Buffered Saline). Since the activation of Integrin *α*_*IIb*_ *β*_*3*_ is largely dependent on the generation of Ca^2+^, nonspecific binding of fibrinogen to integrin *α*_*IIb*_ *β*_*3*_ was assessed in the presence of calcium chelator, EGTA (1 mM). The fluorescence of each platelet sample was analyzed using FACS Calibur cytometer (BD Biosciences, San Jose, CA, USA), (Becton Dickinson Immunocytometry Systems, San Jose, CA, USA).

### Measurement of cAMP

Washed platelets (3 × 10^8^/mL) were pre-incubated for 2 min with PM21 (200 and 100 μg/mL) or aspirin (50 μg/mL) in the presence or absence of 50 μg/mL IBMX (3-isobutyl-1-methylxanthine). 0.1% (*v/v*) DMSO was used as a vehicle. Then platelet aggregation was induced by adding 5 μg/mL collagen in the presence of *Ca* ^2+^ (1 mM) for 5 min. The aggregation reaction was terminated by adding equal volumes of 80% ice-cold ethanol. The samples were then centrifuged at 2000 x *g* at 4 °C for 10 min, and cAMP level of supernatants was determined with cAMP EIA Kit (Ann Arbor, MI, USA).

### Measurement of thromboxane B_2_ (TXB_2_) formation

Washed platelets were pre-incubated with experimental samples and stimulated for aggregation reaction as previously described in this paper. The reactions were terminated by adding ice-cold 2.5 mM EGTA and 100 μM indomethacin. After centrifugation at 12000 x *g* for 3 min at 4 °C, supernatants were collected and TXB_2_ concentration was measured with TXB_2_ EIA kit (Cayman, USA).

### Measurement of serotonin release

Washed platelets were pre-incubated with experimental samples and stimulated for aggregation reaction as previously described. After terminating the aggregation reaction, the mixture was immediately centrifuged at 12000 x g for 5 min at 4 °C. Supernatants were collected and serotonin concentration was measured with serotonin ELISA kit (Labor Diagnostika Nord GmbH & Co, Nordhorn, Germany).

### Measurement of [*Ca*^2+^]*i*

The intracellular concentration of calcium ion [*Ca* ^2+^]*i* was determined with Fura-2/AM as previously described [24]. In this experiment, washed platelets were incubated with 5 mM of Fura-2/AM for 60 min at 37 °C. The Fura-2-loaded platelets (3 × 10^8^/mL) were pre-incubated with experimental samples and stimulated for aggregation reaction as previously described. Fura-2 fluorescence was measured by spectrofluorometer (F-2500, Hitachi, Tokyo, Japan) at the emission wavelength of 510 nm with simultaneous excitation at 340 and 380 nm that changed every 0.5 s. From the spectrofluorometric measurements, [*Ca* ^2+^]*i* was calculated as previously described [8] with the following formula: [*Ca* ^2+^]*i* = 224 nM x (*F* - *F*_min_) / (*F*_max_ - *F*), in which 224 nM is the dissociation constant of Fura-2-*Ca* ^2+^ complex, and *F*_min_ and *F*_max_ represent the fluorescence intensity levels at very low and very high *Ca* ^2+^ concentrations, respectively. *F* represents the fluorescence intensity of the Fura-2-*Ca* ^2+^ complex measured at 510 nm after Fura-2-loaded platelets were pre-incubated with experimental samples and stimulated for aggregation reaction as previously described. In our experiments, *F*_max_ was observed when platelet suspensions containing 1 mM *Ca* ^2+^ were solubilized with Triton X-100 (0.1%), while *F*_min_ was observed when platelet suspensions containing 3 mM EGTA were solubilized with Trion-100 (0.1%).

### Immunoblotting assay for ERKs (extracellular signal-regulated kinases) and PI3 K *(Phosphoinositide 3 kinases)*

Washed platelets were pre-incubated with experimental samples and stimulated for aggregation reaction as previously described. After terminating the reaction, lysates were then prepared by solubilizing and centrifuging platelets in a sample buffer (0.125 M Tris-HCl, pH 6.8; 2% SDS, 2% β-mercaptoethanol, 20% glycerol, 0.02% bromophenol blue, 1 μg/mL phenylmethylsulfonyl fluoride, 2 μg/mL aprotinin, 1 μg/mL leupeptin, and 1 μg/mL pepstatin A). Protein concentration was determined by BCA assay (PRO-MEASURE; iNtRON Biotechnology, Seoul, Republic of Korea). Total cell proteins (30 μg) obtained from platelet lysates were resolved by 10% SDS-PAGE and transferred to nitrocellulose membranes in transfer buffer (25 mM Tris at pH 8.5, 0.2 M glycine, and 20% methanol). The membranes were blocked in TBS-T containing 5% nonfat dry milk and incubated with primary antibody diluted in a blocking solution. The membranes were then probed with antibodies of phospho-ERK2, ERK2, phospho-p38, p38, phosphor-PLCγ2, PLCγ2, phospho-PI3 K (p85), PI3 K and β-actin. The blots were then incubated with the horseradish peroxidase-conjugated secondary antibody. Antibody binding was visualized by enhanced chemiluminescence (iNtRON Biotechnology, Seoul, Republic of Korea).

### In vivo carrageenan-induced mouse tail thrombosis model

Male ICR mice weighing 20 ~ 23 g were purchased from Daehan Biolink Co. Ltd. (Eumsung, Republic of Korea). Six mice were arranged in each experimental group. Mouse tail thrombosis was induced by carrageenan according to the previously reported method [26]. Each mouse was treated with 40 μL (1%) carrageenan (Type I) dissolved in physiological saline by intraplantar injection in the right hind paw. PM21 (75 mg/kg, 150 mg/kg and 300 mg/kg), aspirin (50 mg/kg), clopidogrel (50 mg/kg), or vehicle was orally administered 1 h before carrageenan injection and thereafter for 3 days with 24 h interval. Mice were observed for the formation of thrombosis and thrombus lengths were measured and photographed 1 h after the last treatment.

### Ex vivo platelet aggregation assay with carrageenan-induced mouse tail thrombosis model

Mice were orally administered with experimental samples and mouse tail thrombosis was induced as previously described. Blood was collected 1 h after the last treatment, 72 h after the carrageenan injection. Washed platelets were prepared, and platelet aggregation was induced as previously described.

### Statistical analysis

Data were analyzed by one-way ANOVA, followed by Student’s two tailed-*t*-test to evaluate statistical differences between the treatments and vehicle control. Dunnett’s test was utilized to evaluate statistical differences among the data involved in three or more groups. Data obtained from this experiment expressed as mean value ± SEM (standard error of mean). *P <* 0.05 was considered to be statistically significant.

## Results

### Ex vivo effects of PM21 on platelet aggregation

**Fig. 1:**
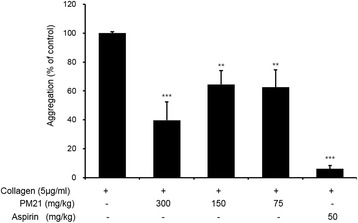
Ex vivo effects of PM21 (a 2:1 mixture of bamboo leaf extract and Japanese apricot fruit extract) on platelet aggregation. Six male rats in each experimental group were treated with test samples. PM21 was orally administered daily after overnight fasting for 3 days to three test groups at the doses of 75 mg/kg, 150 mg/kg, and 300 mg/kg, and aspirin was administered to positive control group at the dose of 50 mg/kg. Blood was collected 1 h after the final treatment. Platelet aggregation was induced by adding 5 μg/mL collagen and terminated after 5 min, and monitored by measuring light transmission with an aggregometer. Data show the mean ± SEM of six measurements. **p < 0.01, ***p < 0.001 versus vehicle control

Ex vivo Inhibition effects of PM21 on platelet aggregation after 3 days of oral administration to SD rats are shown in Fig. 1. The results show that PM21 at the doses of 75, 150, and 300 mg/kg reduces platelet aggregation significantly by 37.4, 35.4, and 60.3%, respectively compared to vehicle control.

### Ex vivo effects of PM21 on ATP release

**Fig. 2:**
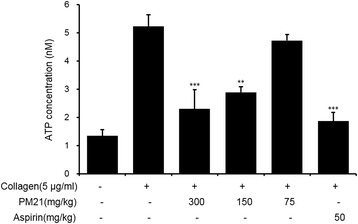
Ex vivo effects of PM 21 on ATP release. Six male rats in each experimental group were treated with test samples. PM21 was orally administered daily after overnight fasting for 3 days to three test groups at the doses of 75 mg/kg, 150 mg/kg, and 300 mg/kg, and aspirin was administered to positive control group at the dose of 50 mg/kg. Blood was collected 1 h after the final treatment. Platelet aggregation was induced by adding 5 μg/mL collagen and terminated after 5 min. ATP release was measured with a luminometer using ATP assay kit. Data show the mean ± SEM of six measurements. **p < 0.01, ***p < 0.001 versus vehicle control

Ex vivo Inhibition effects of PM21 on ATP release after 3 days of oral administration to SD rats are shown in Fig. 2. PM21 at the doses of 150 and 300 mg/kg inhibited ATP release significantly by 44.6 and 55.9%, respectively compared to collagen treated vehicle. The positive control, aspirin (50 mg/kg) significantly inhibited collagen-induced platelet aggregation (Fig. 1) and ATP release (Fig. 2).

### Inhibition effects of PM21 on collagen-induced fibrinogen binding to integrin α_IIb_β_3_

**Fig. 3:**
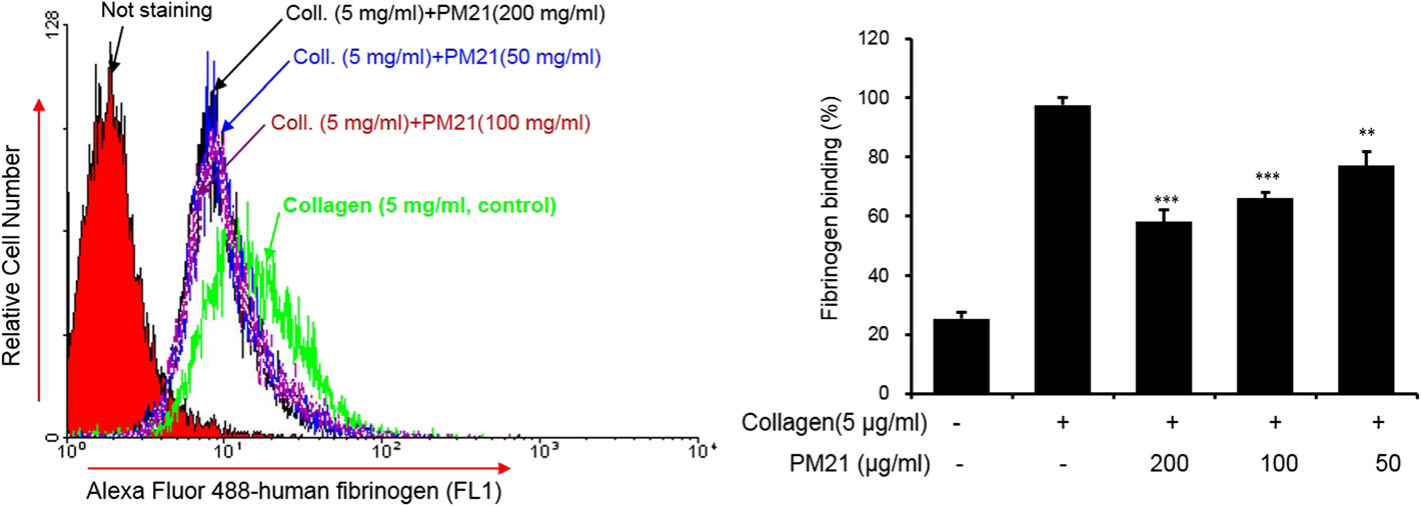
Effects of PM21 on collagen-induced fibrinogen binding to integrin *αIIbβ*_*3.*_ Rat platelets were pre-incubated for 2 min with various concentrations of PM21 (50, 100, 200 μg/mL), then stimulated with 5 μg/mL collagen in the presence of Ca^2+^ (1 mM) for 5 min, and immediately incubated thereafter with fibrinogen Alexa Fluor 488 (20 μg/mL) for 5 min. Nonspecific binding of fibrinogen to integrin αIIbβ3 was assessed in the presence of the calcium chelator, EGTA (1 mM). Fibrinogen Alexa Fluor 488 conjugate binding to platelets was quantified by flow cytometry. Data show the mean ± SEM of at least three independent experiments. ** *p* < 0.01, *** *p* < 0.001 versus vehicle control

As shown in Fig. 3, collagen treatment elevates markedly fibrinogen binding to active integrin *α*_*IIb*_ *β*_*3*_. The results show that fibrinogen binding to integrin *α*_*IIb*_ *β*_*3*_ is significantly reduced in a dose-dependent manner by the treatment of PM21 compared to collagen treated vehicle. PM21 at the doses of 50, 100, and 200 μg/mL reduced fibrinogen binding to active integrin *α*_*IIb*_ *β*_*3*_ significantly by 21.1, 32.4, and 40.6%, respectively compared to collagen treated vehicle.

### Effects of PM21 on cAMP level

Platelets were incubated with different concentrations of PM21 (100 and 200 μg/mL) alone or in combination with IBMX (50 μg/mL), and increase in intracellular cAMP concentration was assessed in resting platelets. The results show that IBMX treatment increases intracellular cAMP level approximately 5.4-fold compared to the untreated vehicle, and PM21 increases intracellular cAMP levels both with and without IBMX compared to each vehicle (Table 1). PM21 increased intracellular cAMP level significantly approximately 9.1-fold and 9.8-fold at the doses of 100 and 200 μg/mL, respectively compared to vehicle control, and PM21 at the presence of IBMX increased intracellular cAMP level significantly approximately 1.9-fold and 2.1-fold at the doses of 100 and 200 μg/mL, respectively compared to IBMX treated vehicle.

**Table 1:** Effects of PM21 on cAMP level, TXB_2_ formation, serotonin release, and intracellular calcium concentration

Treatment (μg/mL)				cAMP level (pmol/10^9^ platelets)	TXB_2_ concentration (pg/mL)	Serotonin concentration (ng/mL)	Intracellular Ca^2+^ concentration (nM)
Collagen	Control	PM21	IBMX				
				0.48 ± 0.12	64.63 ± 63.88	55.25 ± 31.86	139.80 ± 13.70
5	DMSO			–	777.76 ± 55.04	650.46 ± 38.05	2010.70 ± 337.60
5		200		4.71 ± 0.39^***^	347.50 ± 48.76^***^	369.99 ± 18.64^***^	1288.20 ± 310.20
5		100		4.35 ± 0.13^***^	468.93 ± 56.76^**^	476.14 ± 53.98^**^	836.50 ± 88.70^**^
5		50		–	651.07 ± 84.75	358.18 ± 98.73^**^	640.70 ± 153.80^**^
5	Aspirin (50 μg/mL)			6.81 ± 0.73^***^	389.79 ± 17.00^***^	369.99 ± 20.26^***^	632.30 ± 69.20^***^
5			50	2.61 ± 0.16^***^			
5		200	50	5.59 ± 0.25^***^			
5		100	50	4.89 ± 0.59^**^			
5	Aspirin (50 μg/mL)		50	8.11 ± 1.45^**^			

Effects of PM21 on cAMP level, thromboxane B_2_ formation and serotonin release. Rat platelets were pre-incubated for 2 min with PM21 (100 and 200 μg/mL) or aspirin (50 μg/mL) with or without IBMX (50 μg/mL). 0.1% (*v/v*) DMSO was used for vehicle. Then they were stimulated with 5 μg/mL collagen in the presence of *Ca* ^2+^ (1 mM) for 5 min. After termination of aggregation reactions, the samples were centrifuged and cAMP level of supernatants was determined with cAMP EIA Kit. Rat platelets were pre-incubated for 2 min with PM21 (50, 100, 200 μg/mL) or aspirin (50 μg/mL), and stimulated with 5 μg/mL collagen in the presence of *Ca* ^2+^ (1 mM) for 5 min. After termination of aggregation reactions, the samples were centrifuged, and TXB_2_ and serotonin concentrations of supernatants were determined with TXB_2_ EIA kit and serotonin ELISA kit, respectively. Effects of PM21 on intracellular calcium concentration. Rat platelets were incubated with 5 mM Fura-2/AM for 60 min at 37 °C. The Fura-2-loaded platelets were then pre-incubated for 2 min with PM21 (50, 100, 200 μg/mL) or aspirin (50 μg/mL), and stimulated with 5 μg/mL collagen in the presence of *Ca* ^2+^ (1 mM) for 5 min. Fura-2 fluorescence was measured by spectrofluorometer at the emission wavelength of 510 nm. From the spectrofluorometric measurements, intracellular concentration of calcium ion was calculated. Data show the mean ± SEM of at least three independent experiments. *** *p* < 0.001 versus vehicle control

### Effects of PM21 on thromboxane B_2_ formation

The results show that PM21 at the doses of 100 and 200 μg/mL reduces TXB_2_ formation significantly by 39.7 and 55.3%, respectively compared to collagen treated vehicle. PM21 at the dose of 50 μg/mL reduced TXB_2_ formation by 16.3%, however, we obtained no significance (Table 1).

### Effects of PM21 on serotonin release

It is shown in Table 1 that PM21 reduces the level of serotonin release. PM21 at the doses of 50, 100, and 200 μg/mL reduced serotonin concentration significantly by 44.9, 26.8, and 43.1%, respectively compared to collagen treated vehicle.

### Effects of PM21 on intracellular calcium concentration

Collagen increased intracellular concentration of calcium ion up to 2010.7 ± 337.6 nM, which was significantly inhibited by PM21. At the doses of 50 and 100 μg/mL, PM21 inhibited intracellular concentration of calcium ion significantly by 68.1 and 58.4%, respectively compared to collagen treated vehicle (Table 1). However we obtained no significant data with respect to PM21 at the dose of 200 μg/mL.

### Effects of PM21 on phosphorylation of ERK2, p38, PLCγ2 and PI3 K

**Fig. 4:**
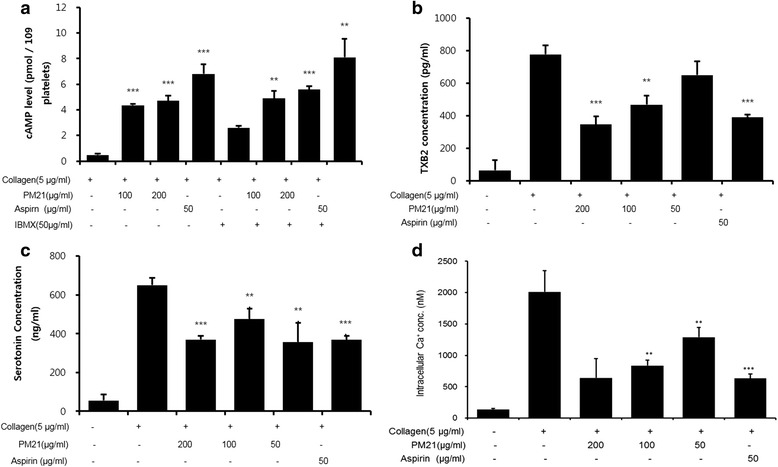
**a** Effects of PM 21 on cAMP level. Rat platelets were pre-incubated for 2 min with PM21 (100 and 200 μg/mL) or aspirin (50 μg/mL) with or without IBMX (50 μg/mL). 0.1% (*v/v*) DMSO was used for vehicle. Then they were stimulated with 5 μg/mL collagen in the presence of *Ca* ^2+^ (1 mM) for 5 min. After termination of aggregation reactions, the samples were centrifuged and cAMP level of supernatants was determined with cAMP EIA Kit. **b** Effects of PM21 on thromboxane B_2_ formation. Rat platelets were pre-incubated for 2 min with PM21 (50, 100, 200 μg/mL) or aspirin (50 μg/mL), and stimulated with 5 μg/mL collagen in the presence of *Ca* ^2+^ (1 mM) for 5 min. After termination of aggregation reactions, the samples were centrifuged and TXB_2_ level of supernatants was determined with TXB_2_ EIA kit. **c** Effects of PM21 on serotonin release. Rat platelets were pre-incubated for 2 min with PM21 (50, 100, 200 μg/mL) or aspirin (50 μg/mL), and stimulated with 5 μg/mL collagen in the presence of *Ca* ^2+^ (1 mM) for 5 min. After termination of aggregation reactions, the samples were centrifuged and serotonin concentration of supernatants was determined with serotonin ELISA kit. **d** Effects of PM21 on intracellular calcium concentration. Rat platelets were incubated with 5 mM Fura-2/AM for 60 min at 37 °C. The Fura-2-loaded platelets were then pre-incubated for 2 min with PM21 (50, 100, 200 μg/mL) or aspirin (50 μg/mL), and stimulated with 5 μg/mL collagen in the presence of *Ca* ^2+^ (1 mM) for 5 min. Fura-2 fluorescence was measured by spectrofluorometer at the emission wavelength of 510 nm. From the spectrofluorometric measurements, intracellular concentration of calcium ion was calculated. Data show the mean ± SEM of at least three independent experiments. ** *p* < 0.01, *** *p* < 0.001 versus vehicle control

**Fig. 5:**
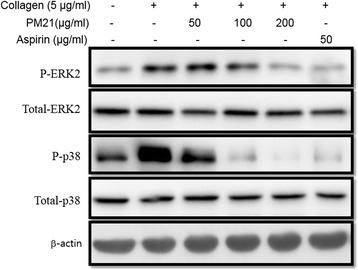
Effects of PM21 on phosphorylation of ERK2 and p38. Rat platelets were pre-incubated for 2 min with PM21 (50, 100, 200 μg/mL) or aspirin (50 μg/mL), and stimulated with 5 μg/mL collagen in the presence of Ca^2+^ (1 mM) for 5 min. After termination of aggregation reactions, total cell proteins were extracted. The proteins were separated by SDS-PAGE and transferred on to nitrocellulose membranes. The membranes were then probed with antibodies against phospho-ERK2, ERK2, phospho-p38, p38 and *β*-actin. Antibody binding was visualized by chemiluminescence. All immunoblots are representatives of three or four independent experiments

**Fig. 6:**
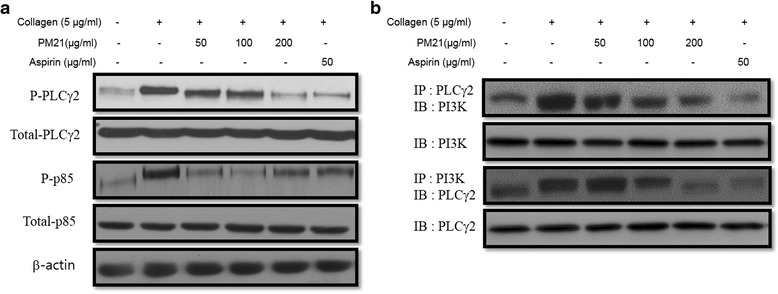
Effects of PM21 on collagen-induced PLCγ2 and PI3 K activation. Rat platelets in the presence of EGTA were incubated with PM21 and then stimulated with collagen. **a** Lysates from the platelets treated with either PM21 (50, 100, 200 μg/mL) or aspirin (50 μg/mL) were immunoblotted to detect the expression levels of PLCγ2 and PI3 K (p85) proteins. **b** Platelet lysates were immunoprecipitated by incubating overnight with anti-PI3 K or anti-PLCγ2, and then further incubated with protein A-Sepharose (PAS). Precipitated proteins were separated by SDS-PAGE and immunoblotted to detect phosphotyrosine residues. Equivalent protein loading was verified by reprobing for PLCγ2 and PI3 K (p85). IP and IB in the diagram represent Immunoprecipitated and Immunoblotted, respectively

Effects of PM21 on phosphorylation of ERK2, p38, PLCγ2, PI3 K, and β-actin in collagen stimulated platelet aggregation were studied and the results are shown in Fig. 4 and Fig. 5a. Phosphorylation of ERK2 and p38 was suppressed by PM21 in a dose-dependent manner in collagen activated platelets whereas β-actin was unaffected (Fig. 5). PM21 also suppressed collagen-induced activation of PLCγ2 and PI3 K (Fig. 6a). Immunoprecipitation assay of PI3 K and PLCγ2 from platelet lysates treated with PM21 was performed. The result shows that PM21 inhibits the expression of PLCγ2 and PI3 K markedly in a dose-dependent manner (Fig. 6b).

### In vivo effects of PM21 on carrageenan induced mouse tail thrombosis

**Fig. 7:**
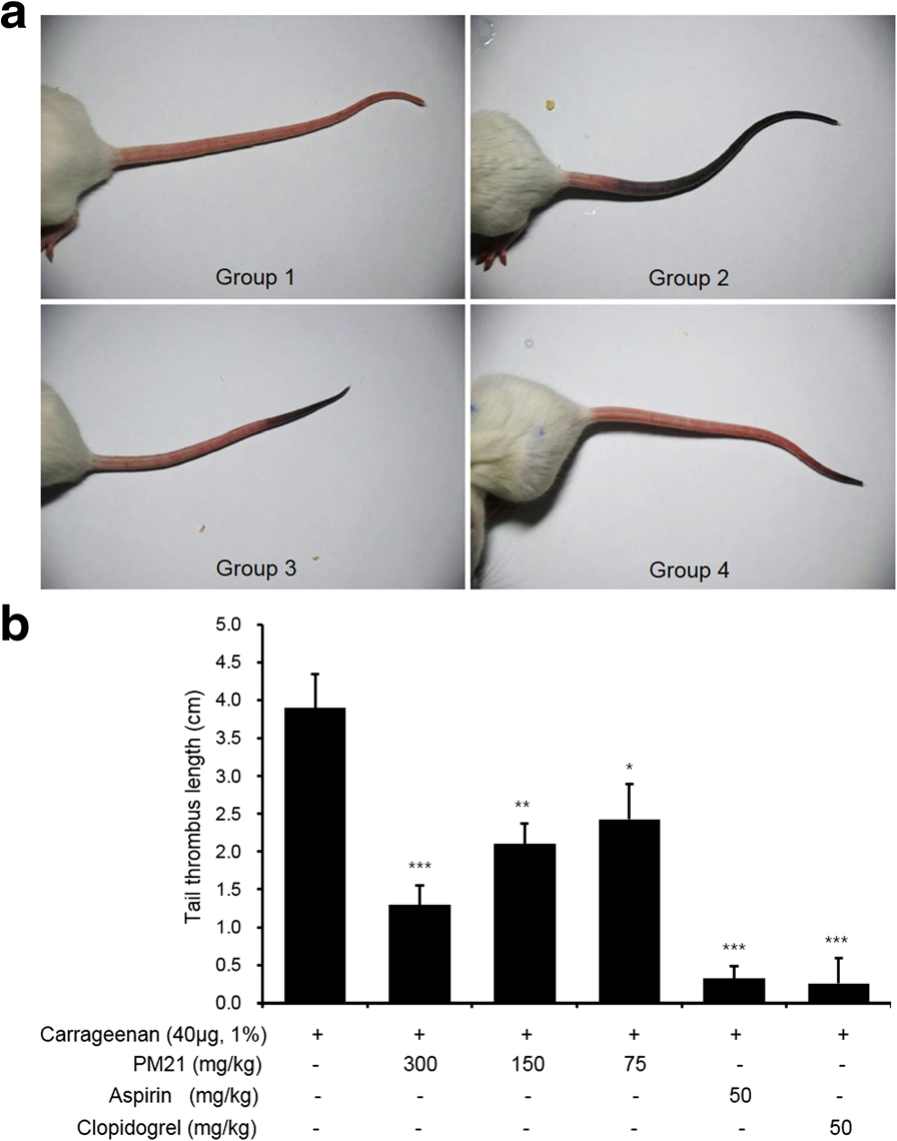
**a** Effects of PM21 on carrageenan induced mouse tail thrombosis. Mouse tail thrombosis was induced by carrageenan. 40 μL (1%) carrageenan (Type I) was administered by intraplantar injection in the right hind paw. PM21 (75 mg/kg, 150 mg/kg, 300 mg/kg), aspirin (50 mg/kg), clopidogrel (50 mg/kg), or vehicle was orally administered 1 h before the carrageenan injection, and thereafter treated with 24 h interval for 3 days. Mice were observed for the formation of thrombosis and photographed 1 h after the last treatment. Group 1 represents normal mouse. Group 2, group 3 and group 4 represent carrageenan treated vehicle, carrageenan plus PM21 (300 mg/kg), and carrageenan plus aspirin (50 mg/kg) treatments, respectively. **b** Effects of PM21 on carrageenan induced mouse tail thrombosis. Mouse tail thrombosis was induced by carrageenan. 40 μL (1%) carrageenan (Type I) was administered by intraplantar injection in the right hind paw. PM21 (75 mg/kg, 150 mg/kg, 300 mg/kg), aspirin (50 mg/kg), clopidogrel (50 mg/kg), or vehicle was orally administered 1 h before the carrageenan injection, and thereafter treated with 24 h interval for 3 days. Mice were observed for the formation of thrombosis and thrombus lengths were measured 1 h after the last treatment. Data show the mean ± SEM of six measurements. * *p* < 0.05, ** *p* < 0.01, *** *p* < 0.001 versus vehicle control

PM21 reduced thrombus formation markedly in carrageenan-induced mouse thrombosis model (Fig. 7a). At the doses of 75, 150 and 300 mg/kg, PM21 reduced the length of mouse tail thrombus significantly by 37.7, 46.2, and 66.7%, respectively in a dose-dependent manner (Fig 7b).

### Ex vivo effects of PM21 on platelet aggregation from carrageenan-induced thrombosis mouse model

**Fig. 8:**
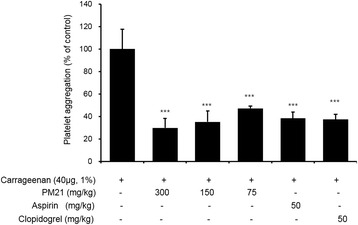
Ex vivo effects of PM21 on platelet aggregation. PM21 (75 mg/kg, 150 mg/kg, and 300 mg/kg) was orally administered 1 h before carrageenan injection, and thereafter three more treatments with 24 h interval for 3 days. Aspirin or clopidogrel was administered to positive control groups at the dose of 50 mg/kg. Blood was collected 1 h after the final treatment. Platelet aggregation was induced by adding 5 μg/mL collagen and terminated after 5 min, and monitored by measuring light transmission with an aggregometer. Data show the mean ± SEM of six measurements. ***p < 0.001 versus vehicle control

The results are shown in Fig. 8. At the doses of 75, 150 and 300 mg/kg, PM21 reduced platelet aggregation significantly by 52.9, 64.7 and 70.2%, respectively in a dose-dependent manner, compared to the vehicle control.

## Discussion

After administration of PM21 to SD rats for 3 days, we performed ex vivo assay to estimate the inhibitory effects of PM21 on platelet aggregation. PM21 inhibited platelet aggregation significantly by 37.4, 35.4, and 60.3% at the doses of 75, 150, and 300 mg/kg, respectively. In order to evaluate in vivo antithrombotic effects of PM21, we used a mouse tail thrombosis model induced by carrageenan. PM21 reduced the length of mouse tail thrombus significantly by 37.7, 46.2, and 66.7% at the doses of 75, 150, and 300 mg/kg, respectively. We also evaluated ex vivo effects of PM21 on platelet aggregation with carrageenan-induced thrombosis mouse model. PM21 reduced platelet aggregation significantly by 52.9, 64.7, and 70.2% at the doses of 75, 150, and 300 mg/kg, respectively. In animal studies, on the whole PM21 has shown significant inhibitory effects on platelet aggregation and thrombus formation. PM21 appears to inhibit ex vivo platelet aggregation more intensely in thrombosis mouse model than in normal rats. It is known that carrageenan induces tissue inflammation and tail thrombosis in animal models [27]. Formation of thrombosis and inflammation are considerably related since inflammation in blood vessels causes thrombosis, on the contrary, thrombus in veins cause inflammation [28]. A component of PM21, *Phyllostachys pubescens* that has been used to treat inflammation in oriental medicine is considered to contribute its anti-inflammatory function to antithrombotic events.

We also evaluated the inhibitory effects of PM21 on major factors involved in platelet activation that leads to adhesion, secretion and aggregation. Collagen was adopted to induce platelet aggregation in our in vitro assays. Binding of collagen to GPVI receptor triggers a signaling cascade that results in the activation of platelet integrins [29]. Activated integrins mediate tight binding of platelets to the extracellular matrix. The dominant integrin on platelet surface is *α*_*IIb*_ *β*_*3*_ that plays a critical role in platelet aggregation [30]. The ligands of *α*_*IIb*_ *β*_*3*_ include fibrinogen, fibronectin and von Willebrand factor [31]. PM21 inhibited significantly fibrinogen binding to active integrin *α*_*IIb*_ *β*_*3*_ proteins in a dose-dependent manner.

cAMP and PKA (cAMP-dependent protein kinase) are important signaling molecules in the regulation of platelet function. Intracellular cAMP induces the activation of PKA, results in the inhibition of platelet aggregation [32]. PM21 increased significantly intracellular cAMP level in collagen induced platelet aggregation assay. It is reported that extracellular ATP regulates platelet reactivity by way of direct action on platelet purinergic receptors or by hydrolysis to ADP [33, 34]. In our ex vivo assay, PM21 inhibited ATP release significantly.

Activated platelets release the stored granule contents such as serotonin and TXA_2_. TXA_2_ formation was monitored by TXB_2_ formation in this study. TXB_2_ release was inhibited by PM21 significantly in this study. Released serotonin enhances platelet aggregation [6, 35]. In our study, serotonin release was inhibited significantly by PM21. Released granule contents from activated platelets lead to a series of downstream events that finally cause to elevate intracellular concentration of calcium ion [9]. In this study, collagen treatment dramatically escalated intracellular calcium concentration and PM21 lowered it in a dose-dependent manner. From the results of serotonin, TXA_2_ and intracellular calcium concentration assays, PM21 shows inhibition potency on the release of granule contents and on the influx of calcium ions from extracellular fluid and mobilization from intracellular pools.

The phosphorylation of signaling molecules such as ERKs is an important step for both outside-in and inside-out signaling that is closely associated with platelet activation and aggregation [36, 37]. GPVI is the major platelet collagen receptor to mediate cellular activation, which is a prerequisite for efficient adhesion, degranulation, and aggregation [8]. Phosphorylated, and hence activated tyrosine kinase Syk initiates a signaling cascade involved in the formation of some effector proteins, most notably PLCγ2 (phospholipase Cγ2) and PI3 K (phosphoinositide-3 kinase). PLCγ2 subsequently induces the formation of second messengers DAG (1,2-diacylglycerol) and IP3 (inositol 1,4,5-trisphosphate). DAG activates protein kinase C, whereas IP3 induces the release of Ca^2+^ from intracellular stores and subsequent Ca^2+^ entry resulting in an increase in intracellular Ca^2+^ concentration [28]. It is also reported that PI3 kinases mediate a critical platelet response involved in affinity regulation of integrin *α*_*IIb*_ *β*_*3*_ [38].

We investigated collagen-induced phosphorylation of ERKs (ERK2, p38, and β-actin) and found that the phosphorylation of ERK2 and p38 was inhibited by PM21 in a dose dependent manner. PM21 also inhibited the phosphorylation and expression of PLCγ2 and PI3 K markedly.

PM21 has shown to affect platelet adhesion by downregulating fibrinogen binding to integrin *α*_*IIb*_ *β*_*3*_ and platelet secretion by reducing the release of dense granule contents, such as ATP, serotonin, TXA_2_, and ionized calcium that are effector molecules to activate further platelet aggregation process. PM21 has also shown to downregulate effector proteins, such as ERK2, p38, PLCγ2, and PI3 K. From experimental results, we recognize that PM21 seems to exert its antiplatelet function to downregulate major events involved in the activation of GPVI receptor and, thereafter, the downstream signaling pathway of activated GPVI.

## Conclusions

PM21 showed remarkable inhibition effects on platelet aggregation and thrombus formation in our animal studies. In antiplatelet mechanism study, PM21 upregulated intracellular cAMP level, and downregulated the release of ATP, thromboxane TXA_2_, and serotonin. It also downregulated intracellular concentration of calcium ion, fibrinogen binding to integrin α_IIb_β_3_, and activation of ERK2, p38, PLCγ2 and PI3 K.

These findings reveal that PM21 exerts its anti-platelet and antithrombotic effects by deactivation of the collagen receptor GPVI signaling pathway and *ERKs* activation pathway as well as inhibition of fibrinogen binding to integrin α_IIb_β_3_.

## Abbreviations

[*Ca*^2+^]*i*: Intracellular calcium ion concentration
ACD: Anticoagulant citrate dextrose
DAG: 1,2-Diacylglycerol
EGTA: Ethylene Glycol Tetra-acetic Acid
ERKs: Extracellular signal-regulated kinases
GPVI: Glycoprotein VI
IBMX: 3-Isobutyl-1-methylxanthine
IP3: Inositol 1,4,5-trisphosphate
MF: *Prunus mume* fruit
PAF: Platelet-activating factor
PI3 K: Phosphoinositide 3 kinases
PKA: cAMP-dependent protein kinase
PL: *Phyllostachys pubescens* leaves
PLCγ2: Phospholipase Cγ2
PM21: a 2:1 mixture of *Phyllostachys pubescens* leaf extract and *Prunus mume* fruit extract
PRP: Platelet-rich plasma
TXA_2_: Thromboxane A_2_
TXB_2_: Thromboxane B_2_
